# Oral Administration of Ginger-Derived Lipid Nanoparticles and Dmt1 siRNA Potentiates the Effect of Dietary Iron Restriction and Mitigates Pre-Existing Iron Overload in *Hamp* KO Mice

**DOI:** 10.3390/nu13051686

**Published:** 2021-05-15

**Authors:** Xiaoyu Wang, Mingzhen Zhang, Regina R. Woloshun, Yang Yu, Jennifer K. Lee, Shireen R. L. Flores, Didier Merlin, James F. Collins

**Affiliations:** 1 Key Laboratory of Precision Nutrition and Food Quality, College of Food Science and Nutritional Engineering, China Agricultural University, Beijing 100083, China; xy.wang@cau.edu.cn; 2 Food Science & Human Nutrition Department, University of Florida, Gainesville, FL 32611, USA; regina.woloshun@ufl.edu (R.R.W.); yuyang2@ufl.edu (Y.Y.); leejennifer@ufl.edu (J.K.L.); srflores@ufl.edu (S.R.L.F.); 3 Center for Diagnostics and Therapeutics, Institute for Biomedical Science, Georgia State University, Atlanta, GA 30303, USA; mzhang21@xjtu.edu.cn (M.Z.); dmerlin@gsu.edu (D.M.); 4 School of Basic Medical Science, Health Science Center, Institute of Medical Engineering, Xi’an Jiaotong University, Xi’an 710049, China; 5 Atlanta Veterans Affairs Medical Center, Decatur, GA 30033, USA

**Keywords:** hepcidin antimicrobial peptide, hereditary hemochromatosis, iron overload, divalent metal-ion transporter 1, *Slc11a2*, nanoparticles, ginger, GDLVs

## Abstract

Intestinal iron transport requires an iron importer (Dmt1) and an iron exporter (Fpn1). The hormone hepcidin regulates iron absorption by modulating Fpn1 protein levels on the basolateral surface of duodenal enterocytes. In the genetic, iron-loading disorder hereditary hemochromatosis (HH), hepcidin production is low and Fpn1 protein expression is elevated. High Fpn1-mediated iron export depletes intracellular iron, causing a paradoxical increase in Dmt1-mediated iron import. Increased activity of both transporters causes excessive iron absorption, thus initiating body iron loading. Logically then, silencing of intestinal Dmt1 or Fpn1 could be an effective therapeutic intervention in HH. It was previously established that Dmt1 knock down prevented iron-loading in weanling *Hamp* (encoding hepcidin) KO mice (modeling type 2B HH). Here, we tested the hypothesis that Dmt1 silencing combined with dietary iron restriction (which may be recommended for HH patients) will mitigate iron loading once already established. Accordingly, adult *Hamp* KO mice were switched to a low-iron (LFe) diet and (non-toxic) folic acid-coupled, ginger nanoparticle-derived lipid vectors (FA-GDLVs) were used to deliver negative-control (NC) or Dmt1 siRNA by oral, intragastric gavage daily for 21 days. The LFe diet reduced body iron burden, and experimental interventions potentiated iron losses. For example, Dmt1 siRNA treatment suppressed duodenal Dmt1 mRNA expression (by ~50%) and reduced serum and liver non-heme iron levels (by ~60% and >85%, respectively). Interestingly, some iron-related parameters were repressed similarly by FA-GDLVs carrying either siRNA, including ^59^Fe (as FeCl_3_) absorption (~20% lower), pancreatic non-heme iron (reduced by ~65%), and serum ferritin (decreased 40–50%). Ginger may thus contain bioactive lipids that also influence iron homeostasis. In conclusion, the combinatorial approach of FA-GDLV and Dmt1 siRNA treatment, with dietary iron restriction, mitigated pre-existing iron overload in a murine model of HH.

## 1. Introduction

Iron is an essential trace mineral with numerous notable biological functions. Iron homeostasis is unique among the dietary minerals as iron-excretory systems do not exist in humans or other mammals. Modulation of the rate of intestinal transport is thus critical to maintain overall body iron levels. Sophisticated homeostatic regulators of iron absorption have developed through evolutionary time, which collectively influence iron absorption in relation to body iron stores, erythroid demand, tissue oxygenation, and during infection and inflammation [1]. Hepcidin is a principal regulator of iron absorption. This liver-derived, peptide hormone controls serum iron levels by regulating iron efflux from cells that absorb (duodenal enterocytes) and store (reticuloendothelial macrophages) iron [2]. The *Hamp* gene (encoding hepcidin) is transactivated in hepatocytes when iron stores are replete and during inflammation; increased hepcidin suppresses intestinal iron absorption and lowers serum iron. During iron depletion, hypoxia and when erythroid demand is elevated, *Hamp* transcription is downregulated, thus increasing intestinal iron transport and raising serum iron.

Transactivation of *Hamp* is impaired in the genetic disease hereditary hemochromatosis (HH) [3], causing low hepcidin, elevated iron absorption and pathological tissue iron overload [4]. Management of HH is principally by phlebotomy [5]; however, this treatment removes iron along with other essential nutrients and biomolecules from the body (i.e., it is not specific for iron). Moreover, some patients are averse to this clinical procedure. Development of new treatment approaches is thus warranted. Dietary interventions may also impact disease severity in HH, and patients may thus be advised to avoid foods rich in highly bioavailable iron, iron-fortified foods and dietary supplements containing extra iron. For example, the Hemochromatosis Society Netherlands, in their publication entitled, “Dietary Advice in HFE-Hemochromatosis” states that:

“…it is likely that the combination of phlebotomy and diet modifications is the best treatment for hemochromatosis. Changes in nutrition can perhaps reduce the number of required phlebotomies. That this preventive method for iron overload gets virtually no attention is quite remarkable because–although (almost) no research is done–it is expected that bloodletting also has an impact on other health aspects, such as the vitamin status of patients”.

Moreover, a recent systematic review on this topic concludes that:

“Despite the limited quantitative evidence and the lack of randomized, prospective trials, dietary interventions that modify iron intake and bioavailability may affect iron accumulation in HH patients. Although this measure may be welcome in patients willing to contribute to their disease management, limited data exist on the clinical and quality-of-life benefit.” [6].

Additional experimental validation of nutritional interventions that may mitigate iron loading in HH is clearly worthy of further consideration.

Since absorption of dietary and supplemental iron is inappropriately elevated in HH, one logical therapeutic intervention involves targeting (and silencing) the main intestinal iron transporters, including: divalent metal-ion transporter 1 (Dmt1) (the main importer of dietary non-heme [or inorganic] iron) and ferroportin 1 (Fpn1) (the main iron exporter). The activity of both transporters is inappropriately elevated in HH [7,8,9]. Low hepcidin increases Fpn1 protein levels on the enterocyte basolateral membrane which secondarily causes a paradoxical increase in Dmt1 protein levels on the brush-border membrane. The latter likely occurs via intracellular depletion of iron (due to high Fpn1-mediated iron export), which stabilizes the Dmt1 transcript via the IRE/IRP iron-regulatory system [10], thus increasing protein expression levels. We previously demonstrated that in vivo knock down of intestinal Dmt1 prevented iron loading in a weanling mouse model of early onset (type 2B) HH (i.e., *Hamp* KO mice). Here, we again focused on Dmt1, but in this case, we sought to test the hypothesis that Dmt1 silencing would mitigate iron loading once already established in older *Hamp* KO mice. We further postulated that Dmt1 knock down would be most effective when combined with dietary iron restriction. Accordingly, we used folic acid-coupled, ginger nanoparticle-derived lipid vectors (FA-GDLVs) [11] to deliver Dmt1 (and negative control) siRNAs to the intestinal epithelium of fully iron-loaded, *Hamp*^−/−^ mice fed a low-iron diet. Subsequent experimental analyses assessed iron status and quantified intestinal iron absorption and tissue distribution.

## 2. Materials and Methods

### 2.1. Preparation of Folic Acid-Conjugated Ginger Nanoparticle-derived Lipid Vectors (FA-GDLVs)

Ginger-derived nanoparticles were isolated according to established protocols [11,12,13,14]. Subsequently, folic acid-conjugated ginger nanoparticle-derived lipid vectors (FA-GDLVs) were fabricated using previously established methods [11]. Folic acid coupling allowed GDLV uptake in the upper small intestine [11], presumably by targeting the proton-coupled folate transporter (PCFT; also named heme-carrier protein 1 [HCP1]), which is highly expressed in the duodenum [15,16]. HPLC-purified, in vivo Dmt1-siRNA (cat. no. 4457308) or negative control siRNA (NC-siRNA) (cat. no. 4457289) (ThermoFisher Scientific) were loaded into FA-GDLVs as described previously [11]. In brief, Dmt1-siRNA or NC-siRNA (3.75 nmol) were mixed with 6 µL of Turbofect in vivo transfection reagent (cat. no. R0541) (ThermoFisher Scientific) and then added to the lipid film of FA-GDLVs under sonication.

### 2.2. Animal Studies

**Figure 1:**
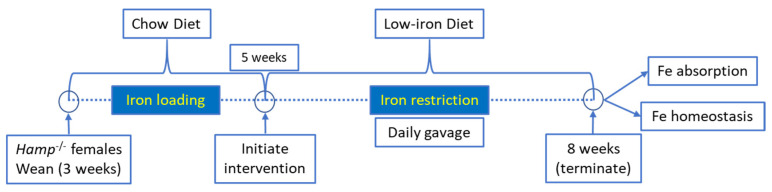
Overview of experimental design.

All animal studies were approved by Institutional Animal Care and Use Committee (IACUC) of the University of Florida. Hepcidin KO (*Hamp*^−/−^) mice on the C57BL/6 genetic background were bred in our animal facility. Female *Hamp*^−/−^ mice were weaned onto a standard chow diet and then, at 5 weeks of age, were switched to low-iron diet (2–6 ppm Fe; Envigo: TD.80396) for the remainder of the experiment (a schematic of the experimental design is provided in Figure 1). These diets contain only inorganic (non-heme) iron. At 5 weeks of age, when consuming a chow diet, these mice are significantly iron loaded, with hepatic non-heme iron levels >20× higher than sex- and age-matched WT mice [11]. Dietary iron restriction was utilized for two reasons: first, we postulated that knock down of Dmt1 would likely be more effective at attenuating absorption when dietary iron was low. This was particularly relevant since the extent of in vivo Dmt1 silencing was uncertain, and modest decreases in Dmt1 activity would likely be more impactful when enteral iron was lower (since the ratio of substrate ions [Fe] to transporter molecules [Dmt1] would also be lower); and second, we sought to assess the impact of dietary iron restriction on the iron-loading phenotype in *Hamp*^−/−^ mice (since this is a logical approach to reduce body iron burden in HH). One day after the dietary switch, mice were given saline (control) or freshly made NC-siRNA or Dmt1-siRNA loaded FA-GDLVs daily (3.75 nmol/dose) by oral, intragastric gavage for 21 days. On each day of the experiment, mice were fasted for three hours before receiving the gavage treatment and then given food immediately thereafter. Water was always provided ad libitum. Our previous studies demonstrated that orally administered GDLVs/siRNAs are non-toxic and non-inflammatory in mice [11,12,17]. At the end of the intervention, half of the mice were euthanized to harvest blood and tissues (liver, spleen, heart, kidney, pancreas, bone marrow and different segments of intestine), and the other half were used for an iron (^59^Fe) absorption study (as described below).

### 2.3. Hematologic and Iron Parameters

Mice were anesthetized by CO_2_ exposure and killed by cervical dislocation. Hemoglobin (Hb) and hematocrit (Hct) levels in whole blood were determined by standard methods [18]. Plasma non-heme iron levels were measured using a colorimetric method [19]. A previously described colorimetric method was used to quantify total iron-binding capacity (TIBC) [19]. Transferrin saturation was calculated as (serum iron/TIBC × 100). Serum ferritin levels were assessed using the Ferritin Mouse ELISA Kit (Abcam, ab157713). Non-heme iron levels in tissues (liver, spleen, heart, kidney, and pancreas) were determined using a standard acid digestion, chromogen-based colorimetric assay as described before [19]; data were normalized by tissue weight.

### 2.4. Iron Absorption Study

Intestinal iron absorption and body iron distribution in experimental mice were determined after oral administration of ^59^Fe, as previously described [18]. Briefly, mice were provided their final dose of saline, or FA-GDLVs/siRNA, and then three hours later, food was removed from cages (but mice were allowed free access to water during this time). The transport solution consisted of 2.5 μCi ^59^FeCl_3_ (500 μCi; 16.26 mCi/mL, Perkin Elmer) diluted into 0.2 mL of a solution containing 0.5 M ascorbic acid, 0.15 M NaCl and 5 μg of Fe from (NH_4_)_2_Fe(SO_4_)_2_. After the three-hour fast, mice were given the transport solution by oral, intragastric gavage. Mice were then immediately given free access to a standard semi-purified (control) diet (with ~48 ppm Fe) (Envigo; TD.80394) and euthanized 24 h later. The control diet was utilized here as we sought to assess iron absorption when dietary intake was normal (i.e., to increase physiological relevance). The 24-h time point was consistent with our previous studies [18,20], and was chosen as a time when all enteral iron would be absorbed (including the slower phase of iron transport that occurs 12 to ~20 h after dosing) [21] and before significant non-specific iron losses had occurred (e.g., via desquamation of epidermal cells, exfoliation of intestinal epithelial cells, and in urine and sweat). Whole-carcass, blood, and tissue radioactivity was measured with a WIZARD2 automatic gamma counter (Perkin Elmer). Intestinal iron absorption was calculated as follows: radioactivity in the carcass minus radioactivity in the entire gut (esophagus to anus) divided by the amount of radioactivity in the oral gavage solution (×100). Blood and tissue radioactive counts were normalized by volume or weight, respectively.

### 2.5. MPO Assay

A small piece of duodenum was freshly harvested and immediately homogenized with Myeloperoxidase (MPO) Assay buffer. MPO activity was quantified using the MPO Activity Assay Kit (Abcam; ab105136) according to the manufacturer’s instruction. Results are shown as relative MPO activity (normalized by the control group).

### 2.6. Western Blotting

Isolated duodenal enterocytes [22] were homogenized in HEM buffer (20 mM Hepes, 1 mM EDTA, 300 mM mannitol, pH 7.4) plus protease inhibitors. After a slow-speed spin to remove undigested material, the homogenate was then centrifuged at 100,000× *g* for 30 min at 4 °C. The resulting pellet was then resuspended in HEM buffer plus protease inhibitors. Protein concentrations were quantified using the Pierce BCA protein assay kit (ThermoFisher Scientific; Waltham, MA). Thirty micrograms of protein were loaded into each lane of 8% polyacrylamide gels. Subsequent to gel running, proteins were transferred to PVDF membranes and then blocked in Odyssey blocking buffer (Licor; Chattanooga, TN, USA). Membranes were then incubated with rabbit anti-DMT1 primary antibody (1:2000) (kindly provided by Dr. François Canonne-Hergaux; French National Institute of Health and Medical Research (INSERM), Digestive Health Research Institute (IRSD), Toulouse, France), mouse anti-FPN1 primary antibody (1:500) (kindly provided by Dr. Mitchell Knutson, University of Florida) or mouse anti-Na/K ATPase antibody (after stripping the membranes) (1:500; cat. no. sc21712; Santa Cruz Biotechnology; Dallas, TX, USA). Blots were then incubated with IRDye 800CW donkey anti-rabbit secondary antibody (1:10,000; cat. no. 925-32213; Licor) or IRDye 680 donkey anti-mouse secondary antibody (1:10,000; cat. no. 926-68072; Licor). Blots were subsequently imaged and protein bands were quantified using a Licor Odyssey CLx immunofluorescent instrument (model- 9141-02V). DMT1 and FPN1 immunoreactive protein band intensities were then normalized to band intensities of Na/K ATPase.

### 2.7. Quantitative Real-Time PCR

Total RNA was isolated by RNAzol^®^ RT reagent (Molecular Research Center, Inc.; Cincinnati, OH) following the manufacturer’s recommended protocol. A Nanodrop spectrophotometer was used to measure RNA concentration and assess RNA integrity. SYBR-Green qRT-PCR was performed based on a well-established protocol [23,24]. The expression of experimental genes was normalized to expression of the housekeeping gene Cyclophilin A (CypA). Primer sequences are listed in Table 1.

### 2.8. Statistical Analyses

Results are depicted as box plots displaying the minimum, the lower (25th percentile), the median (50th percentile), the upper (75th percentile), and the maximum ranked sample. The mean value is indicated by a ‘+’ sign. Statistical analysis was performed using GraphPad Prism (v 8.0) software. Prior to data analysis, the Brown–Forsythe test was used to confirm equal variance. Data were then analyzed by one-way ANOVA. If significant main effects were noted, then by a Tukey’s multiple comparisons test was also performed to assess differences between individual groups. *p* < 0.05 was considered statistically significant.

## 3. Results

### 3.1. FA-GDLVs/siRNA Exposure Had No Detrimental Effects on Experimental Mice

**Figure 2:**
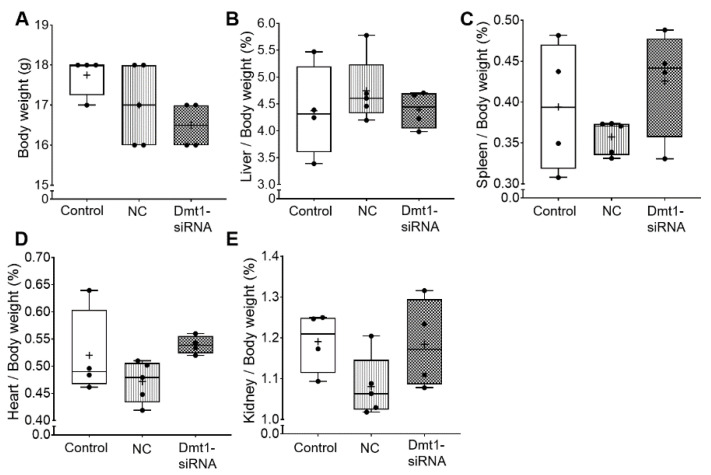
Body weight and tissue weights were not affected by FA-GDLV/siRNA treatments. Body weight (**A**), and relative liver (**B**), spleen (**C**), heart (**D**), and kidney (**E**) weights (as a percentage of total body weight) were quantified in experimental mice. Data are presented as box plots for *n* = 4–5 mice per group. No statistically significant differences were noted between groups (as determined by one-way ANOVA). NC, negative control. +, mean value.

We first sought to determine whether oral administration of FA-GDLVs caused intestinal inflammation or other untoward effects on experimental mice. We did not expect such negative outcomes as previous work has shown that GDLVs and FA-GDLVs are non-toxic and well tolerated by mice [25,26]. Consistent with this, in the current investigation, none of the experimental treatments altered body weights, or the relative mass of the liver, spleen, heart, or kidneys in experimental mice (Figure 2).

**Figure 3:**
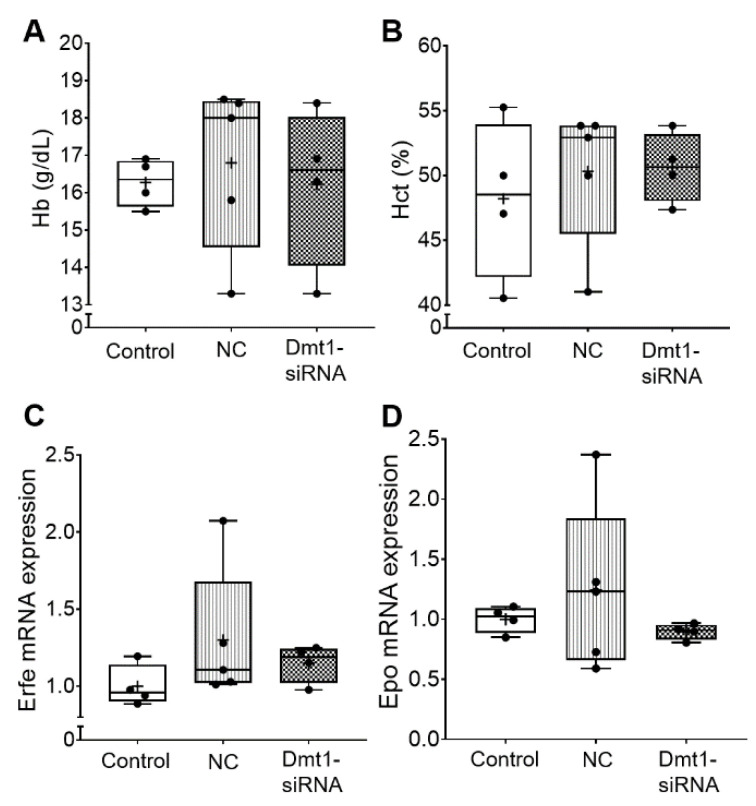
Hematological parameters in the blood and erythropoietic demand were not affected in experimental mice by Fa-GDLVs/siRNA exposure. Blood, bone marrow and kidney were collected and analyzed. Hb (**A**) and Hct (**B**) in whole blood were measured. Erfe mRNA expression in bone marrow (**C**) and Epo mRNA expression in kidney (**D**) were also analyzed. Data are presented as box plots for *n* = 4–5 mice per group. No statistically significant differences were noted between groups (as determined by one-way ANOVA). NC, negative control; +, mean value.

Blood hemoglobin and hematocrit levels were also not altered by the experimental treatments (Figure 3). Consistent with this, biomarkers of erythropoietic demand, including bone marrow erythroferrone (Erfe) [27] and renal erythropoietin (Epo) mRNA expression, were unaffected by FA-GDLVs/siRNA exposure (Figure 3). Note that the magnitude of mRNA expression of Epo and Erfe accurately reflects circulating levels of the functional hormones since both genes are regulated transcriptionally (Erfe probably by Epo/Epo receptor signaling [27], and Epo by hypoxia-inducible *trans*-acting factors [28]).

**Figure 4:**
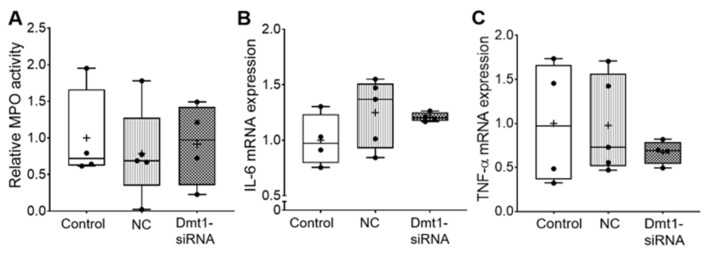
Inflammatory biomarkers did not change after treatments. MPO activity in duodenal epithelial tissue was measured (**A**). IL-6 mRNA expression (**B**) and TNF-α mRNA expression (**C**) in liver were also analyzed. Data are presented as box plots for *n* = 4–5 mice per group. No statistically significant differences were noted between groups (as determined by one-way ANOVA). NC, negative control; +, mean value.

Moreover, myeloperoxidase (MPO) activity in duodenal epithelial tissue was similar among all experimental groups, indicating no localized inflammatory response (Figure 4). Supporting this, hepatic IL-6 and TNFα mRNA expression was not different between groups (Figure 4).

Overall, then, no pathophysiologic effects of FA-GDLVs/siRNA exposure were noted, as all measured outcomes did not vary from saline (control) administration. These observations are congruent with previous studies that have exemplified the safe use of orally administered GDLVs and FA-GDLVs for intestinal siRNA delivery [11,12,17].

### 3.2. FA-GDLV-Mediated siRNA Delivery Downregulated Duodenal Dmt1 mRNA Expression

**Figure 5:**
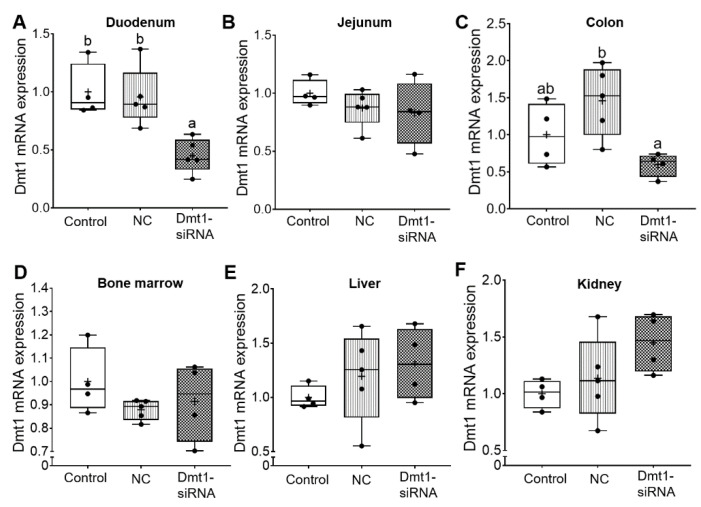
FA-GDLVs loaded with Dmt1 siRNA decreased Dmt1 mRNA expression in duodenum. Female *Hamp*^−/−^ mice were weaned onto a standard chow diet and then switched to a low-iron diet 1 day before NP treatment. Starting at 5 weeks of age, mice were orally gavaged with saline (control), negative control-siRNA (NC-siRNA) or DMT1-siRNA FA-GDLVs daily for three weeks. Dmt1 mRNA expression was quantified in duodenum (**A**), jejunum (**B**), colon (**C**), bone marrow (**D**), liver (**E**), and kidney (**F**). Expression of experimental genes was normalized to expression of Cyclophilin. Data are presented as box plots for n = 4–5 mice per group. Statistical significance was assessed by one-way ANOVA (**A**–**F**), followed by a Tukey’s multiple comparisons test (**A**,**C**). Groups labeled with different letters differ significantly. *p* = 0.0037 (**A**), *p* = 0.0239 (**C**). NC, negative control; +, mean value.

Short-interfering RNAs (siRNAs) are double-stranded RNA molecules that function by RNA interference (RNAi), in which specific transcripts are targeted for degradation [29]. The expected experimental outcome of the current study then would be decreased Dmt1 mRNA expression in tissues/cells targeted by FA-GDLVs when provided by oral, intragastric gavage. As intestinal iron absorption occurs mainly in the duodenum [1], we coupled GDLVs with folic acid to increase uptake in the proximal small intestine [11]. Here, this approach seemed to be effective, as Dmt1 mRNA expression was reduced in the duodenal epithelium (>50%), but no change was seen in jejunum, colon, bone marrow, liver, or kidney (Figure 5). Orally administered FA-GDLVs thus specifically targeted the proximal small intestine, and extra-intestinal effects were not observed. These observations are consistent with our previously published work with FA-GDLVs in mice [11].

### 3.3. Duodenal DMT1 and FPN1 Protein Levels Were Unaffected by FA-GDLV-siRNA Delivery

**Figure 6:**
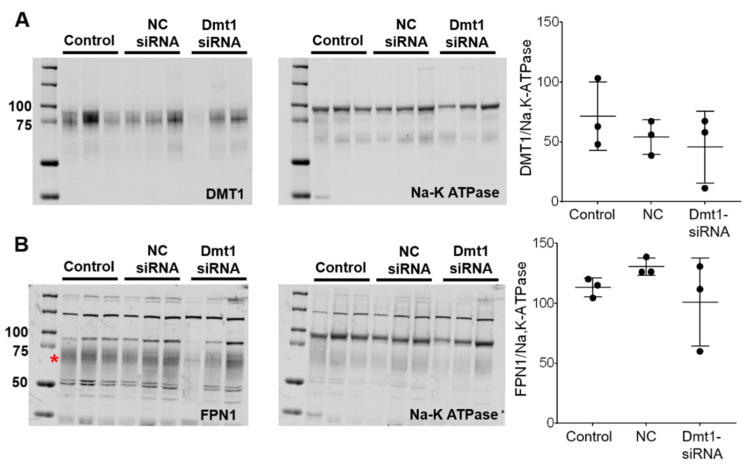
DMT1 and FPN1 protein expression in the duodenal epithelium was unaffected by experimental treatments. Total membrane proteins were isolated from duodenal enterocytes, and equal quantities were run on gels and transferred to membranes. Anti-DMT1 and -FPN1 antibodies were used to detect proteins on the membranes. A Na-K ATPase antibody was used as a control. DMT1 (**A**) and FPN1 (**B**) protein levels were analyzed and normalized by Na-K ATPase protein levels. Quantitative data are mean ± SD for *n* = 3 mice per group and were analyzed by one-way ANOVA (no statistical differences were noted). The asterisk demarcates the full length immunoreactive Fpn1 band (**B**). To aide in interpretation, the entire blots are shown (**A**,**B**). NC, negative control.

siRNA-mediated suppression of Dmt1 mRNA expression would be expected to also decrease DMT1 protein levels, and possibly decrease intestinal iron absorption (since DMT1 is the main iron uptake mechanism in mice) [30]. We thus quantified DMT1 protein levels in experimental mice by Western blot analysis. Also, since iron transfer across the enterocyte basolateral membrane is thought to be the rate-limiting step in mucosal iron transfer [1], it was also important to quantify FPN1 protein expression levels. The DMT1 and FPN1 antibodies we used, which were provided by leading investigators in the iron biology field, have been previously validated for use in mice [31,32,33]. To increase specificity, duodenal enterocytes were isolated (using an established procedure) and total membrane proteins were purified and run on the separating gels. The DMT1 antibody produced single, diffuse bands in each lane of the blots, with the molecular weight of the protein band being in the 75–85 kDa range (Figure 6A), consistent with previous publications [32]. The FPN1 antibody detected several non-specific bands (or lower m.w. degradation products) [33], as well as a diffuse band at ~65–70 kDa which is the immunoreactive FPN1 protein (Figure 6B) (established by using proteins isolated from Fpn1 KO mice; personal communication, Dr. Mitchell Knutson). Using this experimental approach, however, we were unable to detect differences in duodenal DMT1 or FPN1 protein levels between experimental groups of mice (Figure 6). This outcome was unexpected, at least for DMT1, since duodenal mRNA expression was suppressed. High inter-animal variability was apparent, as band intensities varied more than 10-fold (i.e., relative protein expression was >100 in one animal and <10 in another). This clearly contributed to the negative outcome. Nonetheless, since immunoreactive protein on an immunoblot may or may not accurately reflect functional protein levels, it was imperative to quantify intestinal iron absorption in vivo (which should mainly reflect DMT1 activity).

### 3.4. GDLV/siRNA Exposure Decreased Liver and Pancreas Non-heme Iron Content

**Figure 7:**
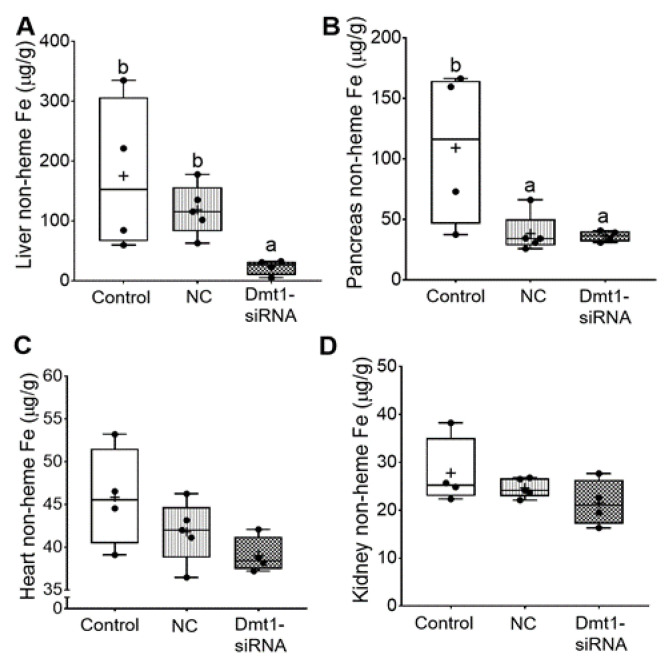
FA-GDLVs/siRNA administration decreased liver and pancreatic iron levels. Shown is non-heme Fe content in liver (**A**), pancreas (**B**), heart (**C**), and kidney (**D**). Data were analyzed by one-way ANOVA (**A**–**D**), followed by Tukey’s multiple comparisons test (**A**,**B**). Data are presented as box plots for *n* = 4–5 mice per group. Groups labeled with different letters differ significantly. *p* = 0.0048 (**A**), *p* = 0.0264 (**B**). NC, negative control; +, mean value.

In this investigation, our experimental approach was to assess the utility of dietary iron restriction combined with attenuation of intestinal DMT1 iron import activity for mitigating iron loading in a murine model of severe HH. To exemplify how the low-iron diet impacted body iron burden, we compared the current data to our previously published data on 7–9-week-old female *Hamp* KO mice (and littermate controls) fed a chow diet with ~200 ppm Fe [11]. In the current study, dietary iron restriction reduced liver iron levels from >1250 µg/g (in chow fed *Hamp* KO mice) [11] to ~150 µg/g. With concurrent knockdown of intestinal Dmt1, liver iron content was reduced to 25–30 µg/g (Figure 7A), which is close to the ~40 µg/g which was previously documented in age- and sex-matched WT mice [11]. In pancreas, iron restriction reduced non-heme iron levels by ~2.5-fold (from ~300 µg/g to 125 µg/g), and FA-GDLV (plus either siRNA) exposure further decreased levels to ~35 µg/g, which is closer to WT values of ~10 µg/g (Figure 7B). In heart (Figure 7C), low-iron intake reduced non-heme iron levels from ~110 µg/g (in chow fed *Hamp* KO mice) [11] to ~40 µg/g, which was closer to WT values (i.e., ~25 µg/g), with FA-GDLV/siRNA exposure having no additional effect. Moreover, dietary iron restriction reduced renal non-heme iron levels from ~125 µg/g (in *Hamp* KO mice) to values typically seen in age- and sex-matched WT mice (i.e., 20–25 µg/g) (Figure 7D) [11]. Renal non-heme iron content was not further reduced by experimental interventions. Finally, spleen non-heme iron content was uniformly low in all mice (20–25 µg/g), with FA-GDLV exposure having no further effects. These values are ~50% lower than in age- and sex-matched mice from our previous study (i.e., 50–60 µg/g) [11]. Spleen iron depletion has been previously described in *Hamp*^−/−^ mice, which is presumably due to high Fpn1-mediated iron export from splenic macrophages (which store and recycle iron obtained by erythrophagocytosis) [34].

In sum, dietary iron restriction reduced hepatic non-heme iron levels in *Hamp*^−/−^ mice, but concurrent knockdown of Dmt1 was necessary to bring values into the same range as in WT controls. In other tissues, low iron intake reduced tissue iron levels closer to WT levels, with FA-GDLV administration potentiating the effect of the low-iron diet only in pancreas (but not in heart or kidneys). Silencing intestinal Dmt1 is thus effective at reducing hepatic iron stores, while FA-GDLV/NC siRNA exposure reduced pancreatic iron loading. The latter observation suggested that ginger contains bioactive lipids [35] that influence iron absorption and homeostasis, since it is highly unlikely that the effects were due to the NC siRNA (which, according to the manufacturer, has no significant sequence similarity to mouse, rat, or human gene sequences, and has been shown to have minimal influences on global gene expression as determined by microarray analysis). This outcome was unanticipated, as FA-GDLV/NC siRNA administration was without effect in our previous study [11].

### 3.5. Dmt1 Knock Down Reduced Parenchymal Iron Loading and Serum Non-heme Iron Levels

**Figure 8:**
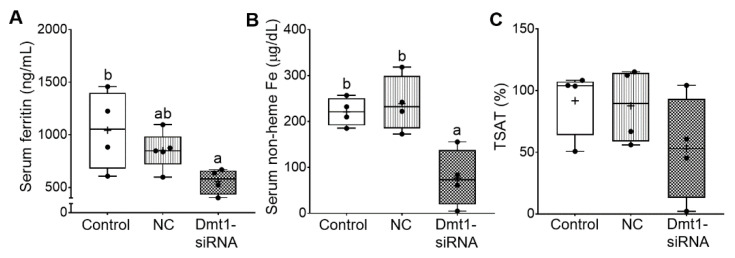
FA-GDLVs/DMT1 siRNA treatment decreased whole body and serum iron content. Shown are serum ferritin levels (**A**), serum non-heme Fe content (**B**), and blood TSAT (**C**). Data were analyzed by one-way ANOVA (**A**–**C**), followed by Tukey’s multiple comparisons test (**A**,**B**). Data are presented as box plots for *n* = 4–5 mice per group. Groups labeled with different letters differ significantly. *p* = 0.0433 (**A**), *p* = 0.0036 (**B**). NC, negative control; +, mean value.

Additional experiments were carried out to quantify biomarkers of iron status, including serum ferritin and non-heme iron, TIBC and blood transferrin saturation (TSAT). In the absence of inflammation or malignant disease, serum ferritin is a reliable indicator of body iron stores [36]. Moreover, increases in serum non-heme iron, and consequent increases in transferrin saturation, typify iron-loading disorders like HH. Successful mitigation of iron loading would thus best be exemplified by reductions in serum ferritin, serum non-heme iron, and TSAT. In our previous study [11], serum ferritin was reported to be ~1000 ng/mL in 7-week-old female *Hamp*^−/−^ mice. Here, control (i.e., saline gavage) mice had similar serum ferritin levels of ~1000 ng/mL, indicating that dietary iron restriction did not decrease these levels. FA-GDLVs/NC siRNA treatment reduced serum ferritin by ~20%, while FA-GDLVs/Dmt1 siRNA exposure further reduced it (by ~50%) to just over 500 ng/mL (although the difference between the treatment groups was not statistically different) (Figure 8A). In these mice (with no inflammation), a 50% reduction in serum ferritin should reflect a reduction in body iron content of a similar magnitude. Serum non-heme iron was also reduced by low-iron feeding (from ~310 µg/dL in chow fed *Hamp*^−/−^ mice [11] to ~220 µg/dL) (Figure 8B), with Dmt1 siRNA administration further reducing it to ~60 µg/dL. TIBC was not different among experimental groups. Moreover, TSAT trended lower in Dmt1 siRNA-exposed mice, but statistical significance was not achieved due to high inter-animal variability; in fact, TSAT values varied 50–100-fold (from 100% to 1–2%) (Figure 8C). Nonetheless, these data demonstrate that GDLVs/Dmt1 siRNA treatment decreased body iron burden in *Hamp*^−/−^ mice.

### 3.6. FA-GDLVs/Dmt1 siRNA Administration Blunted Intestinal Iron (^59^Fe) Absorption and Altered Tissue Iron Distribution

**Figure 9:**
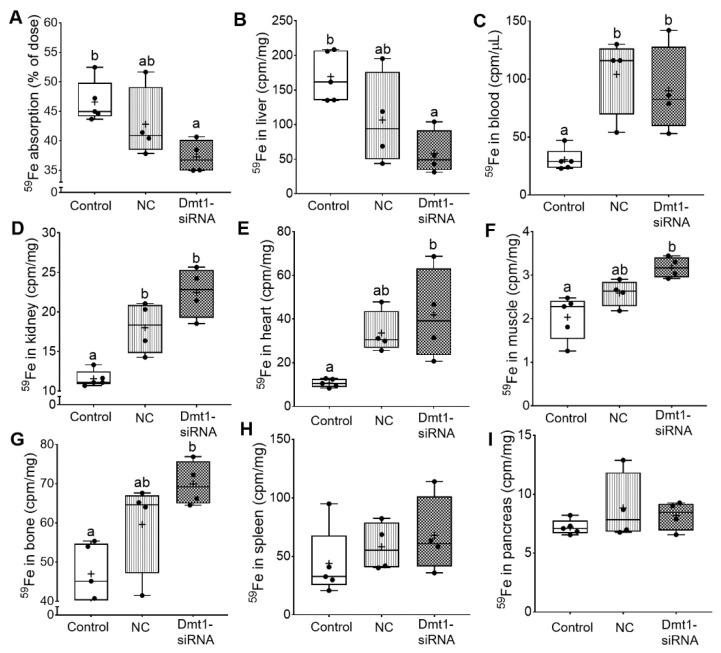
FA-GDLVs loaded with DMT1 siRNA decreased iron absorption in *Hamp*^−/−^ mice. ^59^Fe absorption (**A**), and ^59^Fe radioactivity in liver (**B**), blood (**C**), kidney (**D**), heart (**E**), muscle (**F**), bone (**G**), spleen (**H**), and pancreas (**I**) was measured. Data were analyzed by one-way ANOVA (**A**–**I**), followed by Tukey’s multiple comparisons test (**A**–**G**). Data are presented as box plots for *n* = 4–5 mice per group. Groups labeled with different letters differ significantly. *p* = 0.0276 (**A**), *p* = 0.0161 (**B**), *p* = 0.0064 (**C**), *p* = 0.0003 (**D**), *p* = 0.0103 (**E**), *p* = 0.0041 (**F**), *p* = 0.0089 (**G**). NC, negative control; +, mean value.

So far, we have demonstrated that FA-GDLV/Dmt1 siRNA treatment suppressed Dmt1 mRNA expression in duodenum (by ~50%) and decreased non-heme iron levels in serum (by ~50%) and liver (by ~85%). Serum ferritin and pancreatic non-heme iron were reduced (by ~50%) in mice receiving FA-GDLVs carrying either siRNA (i.e., NC or Dmt1). Given these observations, the next obvious step was to assess intestinal iron absorption. A well-established, oral gavage, radiotracer approach was thus utilized. *Hamp* KO mice have elevated intestinal iron absorption [37], presumably due to high expression/activity of the intestinal iron transporters DMT1 and FPN1. Consistent with this, control *Hamp* KO mice in the current investigation absorbed >45% of the radioactive iron dose, indicative of elevated iron absorption (since WT mice typically absorb 10–15% [or less] of the dose) [38]. Iron absorption decreased by ~10% in mice treated with FA-GDLV/NC siRNA and ~20% in mice exposed to FA-GDLV/Dmt1 siRNA (Figure 9A). ^59^Fe activity in liver was also reduced in both FA-GDLV treatment groups (NC, ~40%; Dmt1, ~70%) (Figure 9B). Surprisingly, FA-GDLV/Dmt1 siRNA treatment increased ^59^Fe activity in heart, muscle, and bone, while both experimental treatments increased ^59^Fe activity in blood and kidney (Figure 9C–G). ^59^Fe activity in spleen and pancreas was not influenced by any of the treatments (Figure 9H, I). These results demonstrated specific effects of Dmt1 siRNA and also direct effects of FA-GDLVs, again suggesting that ginger contains bioactive lipids that can influence iron homeostasis (likely by modulating the magnitude of intestinal iron absorption).

## 4. Discussion

Inappropriately elevated intestinal iron absorption, due to impaired hepatic *Hamp* expression, underlies iron loading in HH. Blocking iron absorption is thus a plausible therapeutic intervention. Targeting intestinal Dmt1 has been previously considered, as small molecule inhibitors have been identified [39,40,41,42,43,44]. To our knowledge, however, demonstration of the effectiveness of Dmt1 blockers in vivo in models of iron-related disease has not been reported to date. We previously demonstrated that FA-GDLV/Dmt1 siRNA treatment prevented iron loading in weanling *Hamp*^−/−^ mice [11], providing proof-of concept that Dmt1 knock down can blunt iron absorption and alter body iron levels. Here, we sought to extend this previous investigation to include the more relevant pathophysiological state of pre-existing iron overload.

We previously established that GDLVs [25,26] and FA-GDLVs are non-toxic to cells and non-inflammatory [11]. Assays used to establish this included biocompatibility assessments using an MTT cell viability assay in mouse colon-26 cells and electrical cell-substrate impedance sensing (which measures real-time barrier function) in Caco2-BBE cells. It was also previously established that FA-GDLVs do not escape the gut when provided by oral gavage, as demonstrated by using a co-administered fluorescent dye and an IVIS in vivo imaging system [11]. Robust fluorescence was detected in the GI tract, but none was observed in kidney, spleen, lung, liver, or heart. Finally, there was no increase in myeloperoxidase (MPO) activity in duodenal tissue extracted from experimental mice treated with FA-GLDVs, indicating a lack of an inflammatory response [45]. Consistent with lack of toxicity, in the current study, body and relative organ weights, myeloperoxidase activity in duodenum, and hepatic IL-6 and TNFα mRNA expression were unaffected by experimental treatments. Blood hemoglobin and hematocrit levels and biomarkers of erythroid demand (i.e., bone marrow erythroferrone and renal erythropoietin mRNA expression) were also unaltered, indicating that Dmt1 siRNA treatment did not cause iron deficiency or decrease iron delivery to the erythron.

As part of this investigation, iron absorption was quantified using a well-established experimental approach. Interpretation of ^59^Fe radiotracer absorption studies can be challenging, since this method offers a ‘snapshot’ of where dietary iron is being distributed during a single 24-h period (and may not reflect total iron accumulation in blood and tissues over a longer period). Since percent iron absorption is calculated from whole body ^59^Fe activity (minus ^59^Fe in the GI tract), reductions in absorption are reflected by decreases in whole-body ^59^Fe accumulation. Mice treated with FA-GDLVs/Dmt1 siRNA assimilated less ^59^Fe from the gavage dose than controls, indicating that vectorial iron transport was reduced. Consistent with this, ^59^Fe activity in the liver was decreased; however, ^59^Fe levels were paradoxically elevated in blood and several tissues tested. The significance of this latter observation is unclear, but nonetheless, iron transfer from the gut to the body was inhibited by FA-GDLV and Dmt1 siRNA treatment.

Our experimental approach here utilized dietary iron restriction (which could improve iron status in HH patients) [6], plus suppression of duodenal Dmt1 expression. We initiated our studies in 5-week-old female *Hamp*^−/−^ mice that were fully iron loaded (i.e., hepatic iron levels are >25-fold higher than in age- and sex-matched WT mice) [11]. Dietary iron restriction reduced body iron levels, with Dmt1 siRNA administration potentiating iron losses. FA-GDLV/Dmt1 siRNA treatment reduced Dmt1 mRNA expression in duodenum (by >2-fold) and decreased intestinal iron absorption by ~20% while ^59^Fe activity in liver was reduced by ~3-fold. FA-GDLVs carrying the NC siRNA also influenced many of the iron-related parameters reported here (although typically, the magnitude was less than the effects when Dmt1 siRNA was included). Mitigation of iron loading in *Hamp*^−/−^ mice was thus most likely due to suppression of duodenal Dmt1 iron import activity and a concomitant reduction in iron transfer to the portal blood circulation. We say this is most likely based upon two facts: (1) DMT1 is the predominant (and probably the only) transporter of enteral non-heme iron in mice (which is the only form of iron present in mouse chow). This is nicely exemplified by the phenotype of the intestine specific Dmt1 KO [30]. These mice are severely anemic and die prematurely at about 6 months of age due to severe iron depletion (i.e., blood hemoglobin levels are lower than most other rodent models of iron deficiency [<5 g/dL; normal is 14–15 g/dL]). It was proposed that the only reason these mice live for ~6 months is due to hepatic iron stores present at birth, and/or minimal absorption of dietary iron due to mosaicism of the Dmt1 KO (as has been documented when using the villin-CRE conditional KO system); and (2) siRNA delivery via oral administration of GDLVs has been shown to specifically target the intestinal epithelium, with no escape of the nanoparticles from the gut [12]. Additionally, in the current investigation, iron absorption may also have reduced by unidentified bioactive components contained within the FA-GDLVs (which may or may not have directly influenced DMT1-mediated iron import).

## 5. Conclusions

This investigation has demonstrated that intestinal Dmt1 is a valid therapeutic target to mitigate iron loading once already established in a murine model of early-onset, severe HH. Attenuation of duodenal Dmt1 expression and inhibition of vectorial iron transfer across the intestinal mucosa potentiated the iron-lowering effect of dietary iron restriction. Unique chemical properties of FA-GDLVs may have also contributed to the positive outcomes of this investigation; however, this is currently not clear and requires further investigation. This combinatorial approach of dietary iron restriction and inhibition of intestinal iron absorption could be further developed and utilized as an adjunctive treatment in HH (or other genetic iron-loading disorders), possibly reducing reliance on phlebotomy.

## Figures and Tables

**Table 1:** qRT-PCR Primer Sequence (5′ to 3′)

Dmt1	GTGATCCTGACCCGGTCTATCG	TGAGGATGGGTATGAGAGCAAAGG
Epo	ATGAAGACTTGCAGCGTGGA	AGGCCCAGAGGAATCAGTAG
Erfe	ACTCACCAAGCAGCCAAGAA	TTCTCCAGCCCCATCACAGT
TNF-α	CACAAGATGCTGGGACAGTGA	TCCTTGATGGTGGTGCATGA
IL-6	CTGCAAGAGACTTCCATCCAGTT	AGGGAAGGCCGTGGTTGT
CypA	CTTACGACAAGCAGCCCTTCATG	AGCTGTTTTTAACTCACTGCTGTTGTA

## Institutional Animal Care and Use (IACUC) Statement

All animal experiments were approved the University of Florida IACUC. The ethic approval code is 201909452.
